# An assessment of federal alcohol policies in Canada and priority recommendations: Results from the 3rd Canadian Alcohol Policy Evaluation Project

**DOI:** 10.17269/s41997-024-00889-3

**Published:** 2024-05-13

**Authors:** Elizabeth K. Farkouh, Kate Vallance, Ashley Wettlaufer, Norman Giesbrecht, Mark Asbridge, Amanda M. Farrell-Low, Marilou Gagnon, Tina R. Price, Isabella Priore, Jacob Shelley, Adam Sherk, Kevin D. Shield, Robert Solomon, Tim R. Stockwell, Kara Thompson, Nicole Vishnevsky, Timothy S. Naimi

**Affiliations:** 1 Canadian Institute for Substance Use Research, University of Victoria, Victoria, BC Canada; 2 Mayo Clinic Alix School of Medicine, Rochester, MN USA; 3 Centre for Addiction and Mental Health, Institute for Mental Health Policy Research, Toronto, ON Canada; 4 Dalla Lana School of Public Health, University of Toronto, Toronto, ON Canada; 5 Department of Community Health and Epidemiology, Dalhousie University, Halifax, NS Canada; 6 Faculty of Law, University of Western Ontario, London, ON Canada; 7 St. Francis Xavier University, Antigonish, NS Canada

**Keywords:** Alcohol drinking, Policy, Legislation, Canada, Federal government, Consommation d’alcool, politique, mesures législatives, Canada, gouvernement fédéral

## Abstract

**Objective:**

To systematically assess the Canadian federal government’s current alcohol policies in relation to public health best practices.

**Methods:**

The 2022 Canadian Alcohol Policy Evaluation (CAPE) Project assessed federal alcohol policies across 10 domains. Policy domains were weighted according to evidence for their relative impact, including effectiveness and scope. A detailed scoring rubric of best practices was developed and externally reviewed by international experts. Policy data were collected between June and December 2022, using official legislation, government websites, and data sources identified from previous iterations of CAPE as sources. Contacts within relevant government departments provided any additional data sources, reviewed the accuracy and completeness of the data, and provided amendments as needed. Data were scored independently by members of the research team. Final policy scores were tabulated and presented as a weighted overall average score and as unweighted domain-specific scores.

**Results:**

Compared to public health best practices, the federal government of Canada scored 37% overall. The three most impactful domains—(1) pricing and taxation, (2) marketing and advertising controls, and (3) impaired driving countermeasures—received some of the lowest scores (39%, 10%, and 40%, respectively). Domain-specific scores varied considerably from 0% for minimum legal age policies to 100% for controls on physical availability of alcohol.

**Conclusion:**

Many evidence-informed alcohol policies have not been adopted, or been adopted only partially, by the Canadian federal government. Urgent adoption of the recommended policies is needed to prevent and reduce the enormous health, social, and economic costs of alcohol use in Canada.

**Supplementary Information:**

The online version contains supplementary material available at 10.17269/s41997-024-00889-3.

## Résumé

**Objectif:**

Évaluer de manière systématique les politiques sur l’alcool actuelles du gouvernement fédéral canadien dans le cadre de pratiques de santé publique exemplaires.

**Méthodes:**

Le projet de l’Évaluation des politiques canadiennes sur l’alcool 2022 a évalué les politiques fédérales sur l’alcool dans dix domaines. Ces domaines de politiques ont été pondérés en fonction de preuves sur leurs répercussions relatives, notamment leur efficacité et leur portée. Une échelle d’évaluation descriptive détaillée de pratiques exemplaires a été élaborée et examinée à l’externe. Entre juin et décembre 2022, des données sur les politiques ont été recueillies dans la législation officielle, sur des sites Web du gouvernement et au moyen de sources identifiées comme telles au cours des itérations précédentes du projet de l’Évaluation des politiques canadiennes sur l’alcool. Des personnes-ressources au sein des ministères concernés ont communiqué d’autres sources de données, examiné l’exactitude et le caractère exhaustif de ces données et apporté les modifications nécessaires. Les données ont été évaluées indépendamment par des membres de l’équipe de recherche. Les scores de politiques finaux ont été inscrits dans des tableaux et présentés sous forme d’une moyenne générale pondérée et de scores non pondérés par domaine.

**Résultats:**

Comparativement aux pratiques de santé publique exemplaire, le gouvernement fédéral du Canada a obtenu un score général de 37 %. Les trois domaines susceptibles d’avoir les plus grandes répercussions, à savoir 1) la fixation des prix et la taxation, 2) le contrôle du marketing et de la publicité, et 3) les mesures contre la conduite avec facultés affaiblies, se sont vu attribuer parmi les scores les plus bas (39 %, 10 %, et 40 % respectivement). Les scores par domaine variaient considérablement, allant de 0 % pour les politiques sur l’âge minimum légal à 100 % pour le contrôle de la disponibilité physique de l’alcool.

**Conclusion:**

De nombreuses politiques sur l’alcool reposant sur des preuves n’ont pas été adoptées, ou l’ont été seulement partiellement, par le gouvernement fédéral canadien. Il est urgent d’appliquer les politiques recommandées pour prévenir et réduire les énormes coûts sanitaires, sociaux et économiques de la consommation d’alcool au Canada.

## Introduction

Globally, alcohol remains one of the leading causes of death and disability and is responsible for 3 million annual deaths and 13.5% of all deaths among 20-to-39-year-olds (World Health Organization, 2022a). Alcohol use is causally linked to over 200 health conditions and injuries, including cancer, liver disease, suicide, road injuries, and others, in addition to causing numerous social harms (e.g., sexual violence, interpersonal violence, crime) (World Health Organization, 2022a). In 2020, alcohol use was associated with over 17,000 deaths and 800,000 hospital visits in Canada (Canadian Substance Use Costs and Harms Working Group, 2023). In 2020, alcohol use in Canada cost nearly $20 billion—more than tobacco and opioids combined (Canadian Substance Use Costs and Harms Working Group, 2023). In contrast, government revenues from alcohol sales totaled $13.5 billion in the same year (Statistics Canada, 2023), thus creating a total alcohol deficit of over $6 billion (the equivalent of approximately $0.30 per standard drink sold). Government measures are crucial to create environments and build communities that support reductions in alcohol use and therefore prevent alcohol-caused harms.

Alcohol control policies are the primary modifiable means by which to prevent and reduce per capita alcohol-caused harms (Martineau et al., 2013). These policies can affect: who sells and distributes alcohol (e.g., control systems); affordability of alcohol (e.g., pricing and taxation); where alcohol is sold (e.g., physical availability); who can buy it (e.g., minimum legal age); where alcohol is advertised and how it is portrayed (e.g., marketing and advertising controls, health and safety messaging); where and when alcohol can be consumed (e.g., liquor law enforcement, impaired driving countermeasures); and what supports are available for individuals to reduce alcohol use and harm (e.g., screening and treatment interventions). Decades of evidence from countries worldwide which have implemented or removed such policies have demonstrated that these measures are a cost-effective component of a comprehensive public health strategy (Babor et al., 2022; World Health Organization, 2010). Despite a large international evidence base demonstrating the effectiveness of alcohol policies, implementation is poor and disproportionate to the harms caused by alcohol due to a lack of political will as well as vested corporate interests thwarting government efforts to strengthen alcohol control measures (McCambridge et al., 2018; Saxena, 2018).

Similar to other countries, in Canada, the legislative authority over alcohol policies falls under the purview of both federal and sub-federal governments (Canadian Centre on Substance Use and Addiction, 2019; Smart, 1984). For example, broadcast advertising of alcohol is regulated under federal jurisdiction pursuant to the Canadian Radio-television and Telecommunications Commission (CRTC) code (Government of Canada, 1996). However, in contrast, provincial and territorial (P/T) governments in Canada control most aspects related to the physical retail availability of alcohol, including the trading hours and allowable density of alcohol retail stores and bars (Canadian Partnership Against Cancer, 2021). In some domains of alcohol policies, federal policies can support or incentivize P/T-level policies. For example, while the majority of alcohol pricing regulations are currently set by P/T governments in Canada, the federal government could implement federal tax incentives to encourage P/T governments to adopt policies such as minimum pricing for alcohol (Solomon, 2018; personal communication). Other policy domains, such as alcohol control systems and impaired driving countermeasures, have shared jurisdiction across levels of government. Therefore, effective coordination between levels of government is essential for comprehensive policy changes in these domains.

The Canadian Alcohol Policy Evaluation (CAPE) was developed to provide a comprehensive and systematic assessment of alcohol policy domains in Canada compared with evidence-informed international best practices. Specifically, CAPE evaluates alcohol policy on a range of domains across government sectors engaged in the regulation, distribution, financial supervision, and public health management of alcohol (Giesbrecht et al., 2016). The original CAPE methodology was modelled based on international peer-reviewed evaluations of alcohol policies (Anderson et al., 2009; Babor et al., 2010; Brand et al., 2007), as well as an approach by Mothers Against Drunk Driving Canada to assess impaired driving policies (Solomon et al., 2003, 2018). The first iteration of CAPE evaluated the 10 Canadian provinces across 10 policy domains (Giesbrecht et al., 2013, 2016). The second CAPE iteration additionally evaluated the three territories as well as the federal government (Vallance et al., 2021; Wettlaufer et al., 2019). The third iteration of CAPE evaluated federal and provincial/territorial governments using updated evaluation criteria (e.g., new indicators), based on emerging evidence on alcohol policies. Each iteration of CAPE represents a point-in-time snapshot of the alcohol policies in place at the time of data collection, and while CAPE can monitor alcohol policy changes, updated evaluation criteria (e.g., new indicators, updated weighting) with each iteration means that evaluation scores are not directly comparable across time. The purpose of this paper is to use the results of the 3rd CAPE to characterize current Canadian federal alcohol policies and to assess them in relation to public health best practices.

## Methods

The federal component of the CAPE project assessed alcohol policies across 10 evidence-informed domains; each domain was comprised of related policies that fall under federal jurisdiction. Table 1 lists the 10 federal policy domains and their key assessment criteria. Policy domains were selected based on published evidence on alcohol policies, including both systematic thematic literature reviews and other alcohol policy frameworks (Anderson et al., 2009; Burton et al., 2017; Nelson et al., 2013; World Health Organization, 2010, 2019, 2022b). Assessment criteria were developed based on peer-reviewed publications that outlined procedures for scoring alcohol policies (Anderson et al., 2009; Babor et al., 2010; Brand et al., 2007; Karlsson & Osterberg, 2001; Naimi et al., 2014; Solomon et al., 2018). Detailed information regarding the development of and evidence for policy domains and their assessment criteria is reported elsewhere (Farkouh et al., 2023).

**Table 1:** Key assessment criteria for CAPE federal policy domains, with domains listed in order of weight for effectiveness and scope

Domain	Key components
Pricing and Taxation	• Minimum pricing • Alcohol sales taxes • Alcohol excise taxes • Proportion of ethanol-based volumetric taxation
Marketing and Advertising Controls	• Comprehensiveness of alcohol marketing and advertising restrictions for mass media • Enforcement of marketing and advertising regulations • Monitoring and public reporting of alcohol industry marketing activities
Impaired Driving Countermeasures	• Impaired driving blood alcohol concentration (BAC) limits for the general population • Impaired driving BAC limits for federally regulated professionals • Breath testing legislation • Evidentiary blood samples • Tracking of impaired driving statistics
Health and Safety Messaging	• Status of enhanced alcohol labelling components • Quality of enhanced alcohol labelling components • Comprehensiveness of health and safety messaging
Physical Availability	• Government controls on commercial alcohol imports • Government controls on personal alcohol imports
Control System	• Federal Alcohol Act • Comprehensiveness of Federal Alcohol Act • Federal government control over alcohol • Public health–informed policy decisions
Minimum Legal Age	• Federal legal age for the sale of alcohol • Level of legal age for alcohol sales in federally controlled areas
Alcohol Strategy	• Status of the national alcohol strategy • Comprehensiveness of the alcohol strategy • Implementation of national alcohol strategy
Screening and Treatment Interventions	• National alcohol guidance • Federal funding for screening, brief intervention, and referral (SBIR) initiatives at P/T level • Federal SBIR initiatives • Federal funding for treatment initiatives • Federal treatment initiatives • Federal tracking and reporting of SBIR and treatment initiatives
Monitoring and Reporting	• Comprehensiveness of national alcohol monitoring program • Accessibility of reporting • Leadership for alcohol monitoring and reporting

While all the policy domains play a role in preventing and reducing alcohol harms, they vary in their degree of effectiveness and scope. To account for this, weights were applied to the policy domains by rating them according to their effectiveness and scope. Effectiveness constituted the evidence for the capacity of the policy to reduce harms (score out of 5). Scope constituted the evidence for both the proportion of a population affected by the policy and the proportion of alcohol harms the policy can affect (score out of 5). Higher scores indicated greater effectiveness and broader scope. Domain weights were determined using an iterative Delphi method, a widely used technique to ascertain expert consensus on various topics that is used by leading health organizations, such as the WHO (World Health Organization, 2021a). The Delphi method has been widely applied to various topics in health care, education, and other disciplines (RAND Corporation, (n.d.)) and has demonstrated validity, including for its use in the social sciences (Landeta, 2006; Tomasik, 2010). In our study, each of 13 topic experts anonymously submitted independent ratings for effectiveness and scope for each domain, after which experts met, discussed the ratings, and addressed any discrepancies in scores. Following this, each expert independently submitted their finalized anonymized ratings. The average weight for each domain was calculated by multiplying the average effectiveness score by the average scope score (possible total of 25). Table S1 (Supplementary Material) shows the results of the federal policy domain weighting in rank order from highest (Pricing and Taxation) to lowest (Monitoring and Reporting).

Domains were composed of evidence-informed, best-practice alcohol policy indicators and sub-indicators that were based on previous iterations of CAPE and were selected and weighted via an iterative process involving multiple rounds of team discussions, as well as comments from three international peer reviewers (Farkouh et al., 2023). For example, the Pricing and Taxation domain consisted of indicators related to minimum pricing, alcohol-specific sales taxes, and alcohol excise taxes. Within each indicator were sub-indicators (e.g., for minimum pricing, one sub-indicator was minimum pricing in federally controlled areas such as national parks and military installations). In some cases, practice indicators were assessed (e.g., for pricing and taxation domain, the proportion of all federal alcohol taxes that are based on ethanol content). Certain domains were allocated additional points for synergy of policies and/or penalties for exceptions or loopholes that compromise policy effectiveness. Each domain was assigned a total of 10 points, and points were distributed within each domain by considering the effectiveness and scope of each indicator/sub-indicator, as determined by team meetings and input from peer reviewers.

A detailed data collection template was used to record publicly available information on federal alcohol policies. Data were collected by three research team members (EKF, KV, AW) between June 1 and December 1, 2022; policies that were in place as of December 1, 2022, were included for analysis. Official government sources (e.g., *Food and Drugs Act*, Canada’s *Criminal Code*, Canadian Government websites) were checked first, along with any sources identified from previous iterations of CAPE (see Table 2). If information from these sources was absent or incomplete, an internet browser search with key terms was conducted to identify other potential sources (e.g., online reports). Key contacts within the federal government and related organizations (see Table 2) were identified and asked to provide data not available in the public domain (e.g., Correctional Service Canada was contacted to acquire information about whether treatment services, beyond 12-step peer-to-peer models, are provided to corrections populations). Subsequently, these contacts validated the data by reviewing the accuracy and completeness of the data files and provided additional sources or amendments as needed. These comments were then incorporated into the data for final assessment.

**Table 2:** Data sources and number of validators by policy domain

Domain	Data sources^1^	Validators^2^
Pricing and Taxation	• Canada Revenue Agency website • Excise Tax Act (R.S.C., 1985, c. E-15) • Public Accounts of Canada 2021, Volume II • Statistics Canada	• Canada Revenue Agency (*n* = 1) • Health Canada (*n* = 1)
Marketing and Advertising Controls	• Canadian Radio-television and Telecommunications Commission Code for broadcast advertising of alcoholic beverages • Canadian Food Inspection Agency website • Advertising Standards Canada website	• Canadian Radio-television and Telecommunications Commission (*n* = 1) • Health Canada (*n* = 2) • Advertising Standards Canada (*n* = 1)
Impaired Driving Countermeasures	• Criminal Code (R.S.C., 1985, c. C-46) • Department of Justice Canada website • Traffic Injury Research Foundation website • Statistics Canada • The Alcohol and Drug Crash Problem in Canada, Report by Canadian Council of Motor Transport Administrators (2016)	• Department of Justice Canada (*n* = 1) • Transport Canada (*n* = 1) • Health Canada (*n* = 1)
Health and Safety Messaging	• Food and Drugs Act (R.S.C., 1985, c. F-27) • Canadian Food Inspection Agency website • Health Canada website	• Health Canada (*n* = 1)
Physical Availability	• Importation of Intoxicating Liquors Act (R.S.C., 1985, c. I-3) • Canadian Border Services Agency website	• Canadian Border Services Agency (*n* = 1) • Global Affairs Canada (*n* = 1)
Control System	• Food and Drugs Act (R.S.C., 1985, c. F-27) • World Trade Organization articles: - General Agreement on Tariffs and Trade (GATT 1947) Article XVII:4(a) - General Agreement on Trade in Services (GATS) Article XIV-b - Canada-United States-Mexico Agreement (CUSMA) – Chapter 3 – Agriculture Article 3.C.3.19 • Global Affairs Canada website • Canadian Border Services Agency website • Duty Free Shop Regulations (SOR/86–1072) • Registry of Lobbyists website, Office of the Commissioner of Lobbying of Canada	• Health Canada (*n* = 2) • Canadian Border Services Agency (*n* = 1) • Global Affairs Canada (*n* = 1)
Minimum Legal Age	• The National Alcohol Strategy Monitoring Project: A Status Report (2017)	• Health Canada (*n* = 1) • Department of Justice Canada (*n* = 1) • Transport Canada (*n* = 1)
Alcohol Strategy	• The National Alcohol Strategy Monitoring Project: A Status Report (2017) • Reducing Alcohol-Related Harm in Canada: Toward a Culture of Moderation: Recommendations for a National Alcohol Strategy (2007)	• Health Canada (*n* = 2) • Canadian Centre on Substance Use and Addiction (*n* = 1)
Screening and Treatment Interventions	• Canadian Centre on Substance Use and Addiction website • Health Canada website • Correctional Service Canada website • The Department of National Defence website • Health and Lifestyle Information Survey of Canadian Forces Personnel (2013/2014)	• Health Canada (*n* = 2) • Correctional Service Canada (*n* = 1) • Canadian Forces Health Services Group (*n* = 1)
Monitoring and Reporting	• Health Canada website • Statistics Canada • Public Health Agency of Canada website • Canadian Centre on Substance Use and Addiction website • Canadian Substance Use Costs and Harms Report (2015–2017) • Canadian Institute for Health Information website • Centre for Addiction and Mental Health website • Canadian Institute for Substance Use Research website • Canadian Partnership Against Cancer website	• Health Canada (*n* = 2)

^1^For website sources, specific source webpages within each website are available upon request to the corresponding author

^2^Some validators reviewed data from multiple policy domains, and thus the cumulative number of validators is not equal to the sum of the number of validators per policy domain. There were 12 validators in total as follows: Canada Revenue Agency (*n* = 1), Health Canada (*n* = 2), Canadian Radio-television and Telecommunications Commission (*n* = 1), Advertising Standards Canada (*n* = 1), Department of Justice Canada (*n* = 1), Transport Canada (n = 1), Canadian Border Services Agency (*n* = 1), Global Affairs Canada (*n* = 1), Canadian Centre on Substance Use and Addiction (*n* = 1), Correctional Service Canada (*n* = 1), and Canadian Forces Health Services Group (*n* = 1)

Scoring for each policy indicator and sub-indicator was conducted independently by two of three possible research team members (EKF, KV, AW) who compared data on each existing policy against a pre-defined scoring rubric (see Table S2, Supplementary Material) to determine the number of points it should receive. The scoring for each domain was then reviewed by two additional team members and any discrepancies in scoring were subsequently discussed and resolved. Scores for each domain were calculated by summing the individual indicator and sub-indicator scores, including any bonus or penalty points. The overall federal alcohol policy score was determined by calculating a weighted average across the 10 policy areas according to the domain weighting listed in Table S1 (Supplementary Material). Percentage scores were also converted to letter grades to enhance public comprehension of the results (for percentage to letter grade conversion, see Table S2, Supplementary Material).

## Results

**Fig. 1:**
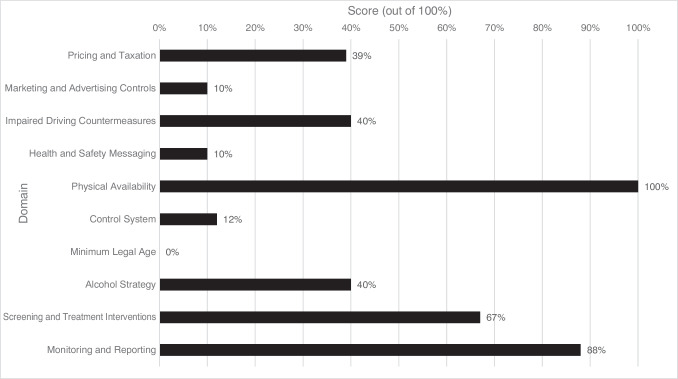
Federal policy domain scores, 2023. Domains are listed in order of their weight, with Pricing and Taxation being weighted with the most evidence of effectiveness and scope

Overall, the Canadian federal government received a total weighted score of 37% (37/100 points) across the 10 policy domains. Figure 1 shows the scores for each policy domain. The following sections provide the results for each domain, ranked in descending order of domain weight (i.e., policy effectiveness and scope). A complete breakdown of scoring by indicator/sub-indicator can be found in Table S2 (Supplementary Material).

### Pricing and Taxation—Score: 39%

Pricing and Taxation was the top ranked domain for its ability to reduce alcohol harms; however, the federal government scored 39% for their pricing and taxation policies. The federal alcohol sales tax rate is currently 5%, which is lower than the achievable rate (as determined by our team of experts) of greater than 12% (Elder et al., 2010). While excise taxes meet recommended targets for high-strength spirits (i.e., $13.04/L pure ethanol), this is not the case for beer, wine, and spirits/coolers (recommended rate of $8.75/L pure ethanol), as excise tax rates for these beverages categories are not based on ethanol content. Further, although there is inflation-based indexation of excise taxes for all alcoholic beverages, there is no unified excise tax rate that is tied precisely to ethanol content, which would ensure that higher-strength alcohol products are taxed at a correspondingly higher rate than lower-strength products (Babor et al., 2010). There is no minimum pricing in federally controlled areas, and the federal government provides no incentives to the P/Ts to implement minimum pricing on alcohol sales.

### Marketing and Advertising Controls—Score: 10%

The federal government scored 10% for its marketing and advertising regulations. There are no restrictions on the total amount of alcohol advertising that can be delivered or its placement (e.g., no restrictions on broadcasting alcohol advertisements in media disproportionately watched by youth). The CRTC code itself has not been updated since 1996 and does not cover digital media advertising. Third-party advertisers (e.g., food delivery applications such as Uber Eats and Skip the Dishes) are permitted to advertise alcohol under federal law. There is no industry-independent mandatory pre-screening of advertisements nor is there an industry-independent enforcement authority or complaint system. There is also no official surveillance or reporting of alcohol industry marketing activities.

### Impaired Driving Countermeasures—Score: 40%

The federal government scored 40% for its Impaired Driving Countermeasures. The federal *Criminal Code* blood alcohol concentration (BAC) limit for driving is 0.08%, versus an ideal of 0.05% (World Health Organization, 2019), and the maximum BAC level for federally regulated commercial drivers is greater than the recommended limit of 0.02% (World Health Organization, 2019). The *Criminal Code* authorizes the police to conduct random roadside breath testing if they are in possession of an alcohol screening device. The *Criminal Code* also empowers police to demand evidentiary blood samples, but only in limited circumstances (e.g., only if police can demonstrate the driver is unable to provide or it is impractical to obtain a breath sample) (Purssell et al., 2010; Solomon et al., 2023).

### Health and Safety Messaging—Score: 10%

Health and Safety Messaging scored 10%. Although it is recommended to have health and safety information on alcohol containers (Canadian Centre on Substance Use and Addiction, 2022; Dimova & Mitchell, 2022; Giesbrecht et al., 2022; Kokole et al., 2021; World Health Organization, 2021b), there are currently no federal regulations mandating alcohol warning labels regarding cancer, pregnancy, youth consumption, impaired driving, or any other issue, on beverage containers. There are also no required labels for standard drink information (i.e., number of standard drinks per container and serving size), calorie/nutritional content, or national drinking guidance. While Health Canada’s website does contain evidence-informed information on alcohol use, it lacks any annual public health campaigns that preclude industry input or that occur outside of the holiday season.

### Physical Availability—Score: 100%

The domain with the highest score was Physical Availability at 100%. All commercial alcohol is required to be imported exclusively by a government authority, and limits on personal imports of alcohol (i.e., duty-free purchases for specific time periods) meet recommendations.

### Control System—Score: 12%

Control System scored 12%. There is currently no federal Alcohol Act in place that would allow for a comprehensive approach to alcohol regulation with a public health focus. The federal government provides no incentives to preserve P/T government control over alcohol distribution, and while trade-law exemptions to protect public health exist, they are not specific to alcohol. Moreover, there is no federal legislation requiring the input or involvement of public health or underrepresented groups (e.g., those with lived or living experience) in alcohol policy. The federal government does have a system of recording alcohol industry lobbying activities via the Lobbyist Registry, which is a centralized online public platform.

### Minimum Legal Age—Score: 0%

The domain with the lowest score was Minimum Legal Age at 0%. There is no federally mandated minimum legal age for the sale of alcohol across all jurisdictions or within federally controlled areas.

### Alcohol Strategy—Score: 40%

Alcohol Strategy scored 40%. There is currently no national alcohol strategy drafted and funded independently of the alcohol industry. The most recent national alcohol strategy, developed in 2007, received no direct funding from the federal government and was partially funded by the alcohol industry. Furthermore, the strategy leadership (National Alcohol Strategy Advisory Committee) included membership from the alcohol industry. There is no official federal endorsement of any alcohol strategy.

### Screening and Treatment Interventions—Score: 67%

Screening and Treatment Interventions scored 67%. There is federal funding available to P/Ts for screening, brief intervention, and referral (SBIR) initiatives, and the federal government itself conducts SBIR and treatment initiatives for both corrections and military populations. However, tracking of SBIR and treatment services is only conducted for military populations. In terms of broader population-level guidance, there are federal funds for the Canadian Centre on Substance Use and Addiction (CCSA) to develop updated national guidance on alcohol and health independent of the industry. At the time of data collection, the most current guidance, which had been in place since 2011, was not formally endorsed.

### Monitoring and Reporting—Score: 88%

The federal government achieved its second-highest score of 88% for Monitoring and Reporting. The federal government provides funding for Health Canada to conduct population-level surveillance and public reporting of various indicators including those related to alcohol consumption and alcohol-attributable morbidity, mortality, crime, costs, and policy changes. However, only three of the six policy indicators—alcohol consumption, attributable mortality, and related crime—are reported annually. CCSA, a federally funded arm’s length agency tasked with addressing alcohol and substance use harms, is responsible for monitoring and reporting of alcohol-related indicators and has conducted knowledge translation activities in the past 2 years. However, there is no accessible, online centralized reporting site for all alcohol indicators.

## Discussion

To our knowledge, this is the first comprehensive and systematic assessment of federal alcohol policy in Canada in the published literature. Alcohol remains a leading preventable cause of health and social problems in Canada. Our results indicate that the federal government is failing to implement many of the most effective evidence-informed alcohol policies. Importantly, the federal government achieved its lowest scores in the policy domains that, according to scientific evidence, are the most effective policies which can reduce alcohol harm (Anderson et al., 2009; World Health Organization, 2013). For example, the top three domains (Pricing and Taxation, Marketing and Advertising Controls, and Impaired Driving Countermeasures) all received failing grades, while the two lowest weighted domains (Screening and Treatment Interventions, Monitoring and Reporting) both achieved passing grades. Further, with respect to the one domain for which the federal government scored 100%, Physical Availability, this was largely due to the limited scope of their controls in this area (i.e., limits on commercial and personal alcohol imports). These scores demonstrate a need for policy development, with strong public health input, particularly in areas that would have the greatest impact on reducing alcohol harm across the Canadian population (e.g., increasing federal alcohol taxes, strengthening advertising restrictions).

The federal government achieved a similar score in the previous CAPE assessment from 2019 (Wettlaufer et al., 2019). While CAPE scores are not directly comparable over time due to methodological differences, it is not surprising that the scores remained low given the federal government’s lack of adoption of high-impact alcohol policies in the intervening years.

The current CAPE federal assessment identifies several policy areas that need to be addressed. The creation of a federal Alcohol Act, supported by a national alcohol strategy developed free of alcohol industry involvement, would facilitate the implementation of a comprehensive and coordinated public health–focused response to addressing alcohol harms by providing governance and a regulatory structure. Given that federal acts for tobacco and cannabis already exist, developing an Alcohol Act would bring greater coherence to the regulation of legal substances in Canada, and serve towards acknowledging and addressing the magnitude of alcohol’s harms. To be effective, an Alcohol Act should address the most effective alcohol control policies, even in the face of strong industry opposition (e.g., tax increases, mandatory standard drink and health labelling of all alcohol products).

There have been several recent opportunities to enact or strengthen alcohol policies where the government has demonstrated inaction. For example, the federal government’s planned inflation-adjusted increase in the alcohol excise tax was reduced from 6.3% to 2%, apparently in response to industry pressure (Woods, 2023). Additionally, federally mandated alcohol warning labels could be enacted as part of an Alcohol Act, if one existed, or by amending the existing *Food and Drugs Act*. Instead, at the present time, warning labels are having to be pursued through a lengthy and uncertain process of introducing a bill in the Senate, namely Bill S-254 (Parliament of Canada, 2021). Furthermore, the former minister of addiction and mental health appeared to favour voluntary industry labelling in lieu of the proposed mandated labels (Canadian Press, 2023). Also, the federal government has opposed the Republic of Ireland’s move to mandate enhanced warning labels on their alcohol containers (World Trade Organization, 2023). Finally, there has been no formal endorsement of the recently updated Canada’s Guidance on Alcohol and Health, published in 2023.

From an international lens, there is substantial room to strengthen Canada’s federal alcohol policies. Compared to Canada, many countries in the Americas, especially those in Latin America, have higher rates of excise taxes on alcoholic beverages (Roche et al., 2023). Additionally, while there is no federal incentive for minimum pricing in Canada, Scotland implemented a minimum unit price across the country and saw reductions in alcohol purchases, along with a 13% decrease in deaths due to alcohol consumption (Iacobucci, 2023; O’Donnell et al., 2019). In terms of alcohol labelling, Ireland and South Korea have both passed legislation requiring labels with cancer warnings on alcoholic products (World Health Organization, 2023). There are also countries, such as Norway and Lithuania, that have instituted bans on alcohol advertising (Scobie et al., 2022). The Canadian federal government can find many examples of effective, evidence-informed alcohol policies around the world. Key opportunities and corresponding rationales for future Canadian federal alcohol policy development are presented in Table 3. A full list of recommendations for each policy domain can be found elsewhere (Naimi et al., 2023).

**Table 3:** Top opportunities for future federal alcohol policy

Key recommendation	Rationale
Implement a federal Alcohol Act, supported by an industry-independent alcohol strategy^1^	A federal Alcohol Act, including some the policies outlined in this table, would allow an overarching legislative framework for preventing alcohol-related harm that could inform the adoption of evidence-informed alcohol policies at all levels of government (Stockwell et al., 2021). Analogous acts already exist for cannabis and tobacco A supporting alcohol strategy can set priorities to ensure a coordinated, evidence-informed response to alcohol-harms (e.g., supporting the development and dissemination of national drinking guidance). Alcohol industry involvement in government activities has been shown to undermine the development and implementation of alcohol strategies (Baumberg & Anderson, 2007; McCambridge, 2012)
Increase the federal sales and excise taxes on alcohol, index taxes to inflation, and base taxes on ethanol content^2^	Increasing the price of alcohol is one of the most effective strategies to prevent alcohol-related harm (World Health Organization, 2010). Alcohol taxation is one cost-effective means to increasing the price of alcohol, especially if the rate of taxation is based on ethanol content and is indexed to inflation (Babor et al., 2010). Increased federal excise taxes (alcohol duty) have been found to reduce alcohol consumption and related harms (Chaloupka et al., 2019)
Incentivize minimum pricing at the P/T level^2^	Evidence from various Canadian contexts has demonstrated that minimum pricing policies decrease alcohol consumption and prevent alcohol-related crime, deaths, and hospital admissions, especially for those of lower income levels (Kilian et al., 2023; Maharaj et al., 2023; Stockwell et al., 2017; Zhao et al., 2013)
Update the federal alcohol advertising code, disallow industry self-regulation, and implement adequate surveillance and enforcement of alcohol marketing violations^3^	Restricting alcohol advertising is one of the most cost-effective policies for preventing alcohol-related harms (World Health Organization, 2011). The Canadian Radio-television and Telecommunications Commission (CRTC) code was last updated in 1996 (Government of Canada, 1996); since then, the alcohol industry has increasingly relied on digital advertising (World Health Organization, 2020), which is not covered in the code. The CRTC code does not currently set restrictions on advertising volume, advertisement placement, or price-based promotions, and the code’s content restrictions are outdated (e.g., it does not prohibit advertisers from promoting an alcoholic product for perceived health benefits, such as low-calorie content). The Pan American Health Organization recommends an industry-independent pre-screening system, an accessible complaint system, and commensurate and escalating penalties for frequent and/or severe violations (Pan American Health Organization, 2018)
Require labelling on all alcohol containers with standard drink, serving size, and health information, including a cancer warning^4^	In 2017–2018, researchers conducted a real-world alcohol warning label experiment in Whitehorse, Yukon (study site) and Yellowknife, Northwest Territories (control site). The labels’ components included a cancer warning, a standard drink label, and a national drinking guideline label (Vallance et al., 2018). The implementation of labels was associated with a 6.3% decrease in retail sales, as well as increased unprompted recall of drinking guidelines and knowledge of the alcohol-cancer association by liquor store customers (Hobin et al., 2020; Schoueri-Mychasiw et al., 2020; Zhao et al., 2020)
Incentivize and support P/Ts to retain and strengthen their alcohol monopolies^5^	From a public health perspective, government monopolies, in which government has control over the wholesale, retail sale and distribution of alcohol, are preferred over privatized retail sale of alcohol. Canadian jurisdictions that replaced government monopolies with partial or full privatization saw an increase in alcohol-related harms (Adrian et al., 1996; Giesbrecht et al., 2021; Stockwell et al., 2009, 2011)
Set a federal minimum legal drinking age, and incentive P/Ts to raise theirs, ideally to the age of 21^6^	Higher drinking ages are associated with fewer harms among youth and reduced heavy drinking in later life. Specifically, a minimum legal drinking age of 21, as is the case in the United States, has been shown to decrease alcohol consumption among youth and prevent alcohol-related road accidents, suicide, homicide, and the development of alcohol/other drug dependence (DeJong & Blanchette, 2014)

^1^From the Alcohol Control System and Alcohol Strategy domains

^2^From the Pricing and Taxation domain

^3^From the Marketing and Advertising Controls domain

^4^From the Health and Safety Messaging domain

^5^From the Alcohol Control System domain

^6^From the Minimum Legal Age domain

### Limitations

There are limitations to our study. First, there is no universally accepted system for weighting alcohol policy domains and indicators because there are few studies that directly compare the efficacy of policies against one another. Likewise, while we used an iterative Delphi survey technique and the best available evidence to inform development of the scoring rubric (Farkouh et al., 2023), there is inherent variation in interpretation of the research literature and the expert opinion of a given team. This is especially important in the context of indicators which do not have a widely accepted gold standard (e.g., ideal alcohol sales tax rates). In addition, while CAPE assessed the presence of federal alcohol policies, it was less able to assess how and to what extent those policies are operationalized in practice. For example, while CAPE captured the existence of legislated funds for alcohol screening, brief intervention, and treatment for federal populations, we could not assess the quality or accessibility of such services.

## Conclusion

The results of the 3rd Canadian Alcohol Policy Evaluation identified several opportunities for the federal government of Canada to strengthen alcohol policies to better align with international, evidence-informed best practices for reducing alcohol’s health, social, and economic harms. The federal government could prevent alcohol harms by adopting federal policies and encouraging evidence-informed alcohol policy at the P/T level. The government should also ensure ongoing evaluation of both federal and provincial/territorial alcohol policies in comparison to public health best practices, using the latest evidence. Taking these actions would reduce alcohol-caused deaths and illnesses, prevent social harms, and lessen the resulting public economic costs of alcohol use in Canada.

## Contributions to knowledge

What does this study add to existing knowledge?

- This is the first comprehensive assessment of Canadian federal alcohol policy in the published literature.
- Our results demonstrate that the Canadian federal government has significant unrealized potential when it comes to implementing effective evidence-informed alcohol policies that would prevent the harms caused by alcohol.
- Our results also demonstrate that current federal alcohol policy priorities are not informed by scientific evidence. That is, the federal government is failing to adopt policies with proven effectiveness and impact, and instead achieved high scores in policy domains with weaker evidence of efficacy and scope.

What are the key implications for public health interventions, practice, or policy?

- If Canada’s alcohol harms are to be reduced and prevented, the federal government must adopt key evidence-informed alcohol control policies (e.g., increasing federal alcohol taxes, strengthening advertising restrictions).
- Creation of a federal Alcohol Act, supported by a national alcohol strategy developed independently of alcohol industry, could set public health–informed alcohol policy priorities. These include setting inflation-indexed volumetric excise taxes that reflect ethanol content; having federal incentives for P/T-level minimum pricing; implementing stricter restrictions on alcohol marketing; mandating alcohol labels with standard drink, serving size, and health information; and setting a federal minimum legal drinking age, among others.

## Supplementary Information

Below is the link to the electronic supplementary material.

Supplementary file1 (DOCX 79 KB)

## Data Availability

Not applicable.
