# Supplementary material for: An assessment of federal alcohol policies in Canada and priority recommendations: Results from the 3rd Canadian Alcohol Policy Evaluation Project
Source: Can J Public Health. 2024 May 13;115(4):640–53. doi: 10.17269/s41997-024-00889-3 (PMC11303602; doi:10.17269/s41997-024-00889-3)
Supplement: Supplementary file 1 — Supplementary file1 (DOCX 79 KB) [file 41997_2024_889_MOESM1_ESM.docx]

Farkouh EK*, Vallance K, Wettlaufer A, Giesbrecht N, Asbridge M, Farrell-Low AM, Gagnon M, Price TR, Priore I, Shelley J, Sherk A, Shield KD, Solomon R, Stockwell TR, Thompson K, Vishnevsky N, Naimi TS. An Assessment of Federal Alcohol Policies in Canada and Priority Recommendations: Results from the 3rd Canadian Alcohol Policy Evaluation Project. *Canadian Journal of Public Health.*

*Corresponding Author:

Elizabeth K. Farkouh

Canadian Institute for Substance Use Research

2300 McKenzie Ave,

Victoria, BC V8N 5M8

(250) 472-5445

[efarkouh@uvic.ca](mailto:efarkouh@uvic.ca)

**Table S1.** CAPE 3.0 federal policy domain weighting, listed in order of weight for effectiveness and scope.

**Table S2.** Scoring rubric for federal alcohol policy. The score received is bolded or listed as a footnote.

**Table S1.** CAPE 3.0 federal policy domain weighting, listed in order of weight for effectiveness and scope.^[[1]](#footnote-1)^

| **Policy Domain** | **Mean**  **Effectiveness**  **[out of 5]** | **Mean**  **Scope**  **[out of 5]** | **Total Impact Weight (effectiveness x scope)**  **[out of 25]**  **(% weight)** |
| --- | --- | --- | --- |
| **1. Pricing and Taxation** | 4.54 | 4.58 | **20.77 (21.90%)** |
| **2. Marketing and Advertising Controls** | 3.38 | 4.04 | **13.67 (14.41%)** |
| **3. Impaired Driving Countermeasures** | 3.27 | 2.88 | **9.43 (9.94%)** |
| **4. Health and Safety Messaging** | 2.58 | 3.54 | **9.12 (9.61%)** |
| **5. Physical Availability** | 2.81 | 3.08 | **8.64 (9.11%)** |
| **6. Control System** | 2.73 | 3.12 | **8.51 (8.97%)** |
| **7. Minimum Legal Age** | 3.19 | 2.31 | **7.37 (7.77%)** |
| **8. Alcohol Strategy** | 2.08 | 3.00 | **6.23 (6.57%)** |
| **9. Screening and Treatment Interventions** | 2.54 | 2.35 | **5.96 (6.28%)** |
| **10. Monitoring and Reporting** | 1.92 | 2.69 | **5.18 (5.46%)** |
| **Sum of total impact weight** | | | **94.87 (100%)** |

Table S2. Scoring rubric for federal alcohol policy. The score received is bolded or listed as a footnote.^[[2]](#footnote-2)^

| INDICATOR/SUBINDICATOR DETAILS | POINT VALUE | SCORING BREAKDOWN |
| --- | --- | --- |
| 1. Pricing and Taxation | 10 |  |
| 1.1 Minimum pricing | 2 |  |
| 1.1a. Financial incentives for P/T minimum pricing  The jurisdiction was scored on whether they make use of financial incentives for implementing a minimum price at the provincial/territorial level. | 1.5 | **0.00= No financial incentives**, 1.50= Financial incentives to encourage minimum pricing implementation at the P/T level. |
| 1.1b. Minimum pricing in federally control areas  The jurisdiction was scored on whether they implement minimum prices for all alcohol sold on federally controlled lands and waters (i.e., parks, military installations, boats owned by Canadian persons or businesses) with the ideal being an indexed minimum unit price (iMUP). | 0.5 | **0.00 = No minimum prices for federal controlled land/waters**,  0.25= Some components of iMUP implemented in federally controlled land /waters, 0.50= iMUP fully implemented in federally controlled land/waters. |
| 1.1c. Federal minimum price loopholes  The jurisdiction was scored on whether they allow for any discounting or minimum pricing loopholes on federally controlled lands/waters. | -0.125 | 0.125 points were deducted for the minimum pricing score if the jurisdiction had any minimum pricing loopholes that undermine minimum prices on federally controlled lands/waters,  **0.00 = No deduction.** |
| 1.2. Alcohol sales taxes | 2.25 |  |
| 1.2a. Level of federal sales tax (ad valorem - % of price)  The jurisdiction was scored on the level of federal sales taxes applied to alcohol (e.g., GST or other ad valorem federal alcohol sales tax). | 2.25 | 0.00 = Federal alcohol sales tax rate < 5%, **1.00 = Federal alcohol sales tax rate between 5-8%,**  1.50 = Federal alcohol sales tax rate between 9-12%,  2.25 = Federal alcohol sales tax rate > 12%. |
| 1.3. Alcohol excise taxes | 5.5 |  |
| 1.3a. Level of excise tax on alcohol products (tax per unit volume)  The jurisdiction was scored on the level of alcohol excise tax the jurisdiction imposes on alcohol products prior to applying the GST rate. The jurisdiction was scored against an ideal of a unified alcohol volumetric rate of $13.04/ L ethanol for spirits and $8.75/L ethanol for non-spirits (e.g., beer and wine) based on a stepped approach for moving towards a unified rate across all beverage types. Ideal rates account for an 85% upward adjustment for lack of indexation between 1991 and 2017. | 2.5 | The score was determined based on the proportion of the actual alcohol excise tax rate against the ideal rates of $13.04/L ethanol for high strength spirits and $8.75/L ethanol for non-spirit and low strength spirit beverages.^[[3]](#footnote-3)^ |
| 1.3b. Ethanol-based volumetric excise tax by beverage category  The jurisdiction was scored on the degree to which the excise tax reflects alcohol content within each major beverage category. | 2.5 | 0.00 = Flat excise taxes (rate per L beverage), A maximum of 2 points for volumetric excise taxes, with no loopholes (e.g., no discounts or exemptions), for beer wine and spirits, weighted to reflect their proportion of sales based on estimated ethanol content by beverage category. In the case of excise tax exemptions or discounts, a score of zero was applied to the proportion of products that would benefit from the discount and exemption. 0.50 additional points were awarded for having the same rate per litre of ethanol applied across all beverage categories.^[[4]](#footnote-4)^ |
| 1.3c. Indexation of excise taxes  The jurisdiction was scored on whether there was indexation of excise taxes for all beverage types. | 0.5 | 0.00 = Excise tax is not indexed, **0.50 = Ad valorem excise tax rate structure (automatically adjusts for changes in price) or flat rate excise tax is indexed annually to CPI within each beverage type.** |
| 1.4. Proportion of ethanol-based volumetric taxation | 0.25 |  |
| 1.4a. Practice Indicator - Ethanol-based volumetric taxation  The jurisdiction was scored on the proportion of all federal alcohol taxes (i.e., excise taxes and GST) that are volumetric versus not. | 0.25 | A maximum of 0.25 points were awarded based on the proportion of federal alcohol taxes collected by volumetric excise versus sales tax or flat excise tax.^[[5]](#footnote-5)^ |
| 2. Marketing and Advertising Controls | 10 |  |
| 2.1. Comprehensiveness of alcohol marketing and advertising restrictions for mass media | 6 |  |
| 2.1a. Ad bans and volume restrictions  The jurisdiction was scored on whether there were advertising bans or restrictions on the volume of alcohol advertising permitted, across all media types (e.g., advertising bans or restrictions on the number of ads or % of ad space occupied by alcohol ads, etc.), regardless of the advertiser (i.e., government or private). | 2.5 | Jurisdiction awarded full points on indicators 1a-1d for a complete advertising ban. If no full ban on alcohol advertising, the following scores for volume restrictions were applied to each of the following media types: +0.50 for full ban or +0.25 for volume restrictions for broadcast media (radio and television), +0.50 for full ban or +0.25 for volume restrictions for online media services (Netflix, etc.), +0.50 for full ban or +0.25 for volume restrictions for internet, +0.50 for full ban or +0.25 for volume restrictions for social media,  **0.00 = No ad bans or volume restrictions for any media type.** |
| 2.1b. Content restrictions  The jurisdiction was scored on content restrictions that go beyond the CRTC code for all media types regardless of the advertiser (i.e., government or private). | 1 | Jurisdiction awarded the following scores for content restrictions that applied to each of the following media types:  +0.25 Broadcast media (radio and television), +0.25 Online media services (e.g., Netflix, etc.), +0.25 Internet, +0.25 Social media,  **0.00 = No content restrictions for any media type.** |
| 2.1c. Placement restrictions  The jurisdiction was scored on restrictions on the placement of advertisements within all media types (e.g., restrictions prohibiting alcohol ads in media where the target audience is under the minimum legal age), regardless of the advertiser (i.e., government or private), in order to minimize alcohol advertisement exposure of priority populations (e.g., youth, individuals in recovery). | 1 | Jurisdiction awarded the following scores for placement restrictions that applied to each of the following media types:  +0.25 Broadcast media (radio and television), +0.25 Online media services (e.g., Netflix, etc.),  +0.25 Internet,  +0.25 Social media,  **0.00 = No placement restrictions for any media type.** |
| 2.1d. Price-based promotions ban  The jurisdiction was scored on restrictions on price-based marketing strategies/promotions, beyond policies prohibiting the advertisement of alcohol below the minimum price (e.g., policies restricting the advertisement of “cheap” drinks or specials), across all media types and regardless of the advertiser (i.e., government or private). | 0.5 | Jurisdiction awarded the following scores for price-based promotion restrictions that applied to each of the following media types: +0125 Broadcast media (radio and television),  +0.125 Online media services (e.g., Netflix, etc.),  +0.125 Internet, +0.125 Social media,  **0.00 = No price-based promotion restrictions for any media type.** |
| 2.1e. Restrictions by advertiser type  The jurisdiction was scored on whether they had restrictions on alcohol advertisements by non-licensees (e.g., third parties not involved in the production, manufacturing, or sale of alcohol including food delivery services). | 0.5 | **0.00 = Third party permitted to advertise alcohol**,  0.50 = Third party not permitted to advertise alcohol. |
| 2.1f. Coverage of alcohol marketing and advertising regulations across advertisers  The jurisdiction was scored on whether their alcohol advertising restrictions (see 2.1 a-d) applied to all advertisers (e.g., government retailers, private retailers and licensees, FOPs, manufacturers and their agents, SOP holders). | 0.5 | 0.00 = No marketing restrictions or marketing restrictions do not apply to all advertisers, **0.50 = Marketing restrictions apply to all advertisers, including government retailers.^[[6]](#footnote-6)^** |
| 2.2. Enforcement of marketing and advertising regulations | 3 |  |
| 2.2a. Mandatory pre-screening of advertising  Jurisdiction was scored on whether they had a mandatory pre-screening process across media types, regardless of the advertiser, that was conducted by a representative independent from the alcohol industry and alcohol sales, for ensuring alcohol advertisements adhere to the regulations. | 1.5 | +0.375 for pre-screening for broadcast media (radio and television), +0.375 for pre-screening for online streaming services,  +0.375 for pre-screening for internet,  +0.375 for pre-screening for social media,  **0.00 = No mandatory pre-screening for any media type.**  Note: total up to a maximum of 1.5 points |
| 2.2b. Enforcement authority over alcohol advertising    The jurisdiction was scored on whether they had a specific health and/or safety-oriented authority, independent from industry and alcohol sales, responsible for enforcement regardless of advertiser (i.e., government or private). | 0.25 | **0.00 = No independent health/safety authority responsible for enforcement,** 0.125 = An independent health/safety authority responsible for enforcement, but some advertisers (e.g., government stores) are exempt,  0.25 = An independent health/safety authority, free from industry, responsible for enforcement with no exemptions.  Note: total up to a maximum of 0.25 points |
| 2.2ci. Independent online complaints system  Jurisdiction was scored on whether the authority had an established independent online system for receiving complaints geared to the lay public, independent from industry and alcohol sales. | 0.25 | **0.00 = No online formal independent complaint process**,  0.25 = Online formal independent complaint process. |
| 2.2cii. Timelines for complaint adjudication  Jurisdiction was scored on timelines for complaint adjudication (e.g., occurring within 30 days). | 0.25 | **0.00 = No defined timelines**, 0.25 = Adjudication timelines do not exceed 30 days. |
| 2.2d. Penalties for advertising violations  Jurisdiction was scored on whether the independent authority has sufficient enforcement powers, including the ability to levy meaningful sanctions that are commensurate with the violation and that escalate with the frequency of the violation. | 0.5 | 0.00 = No penalties, **0.25 = Penalties commensurate with the violations**, 0.50 = Penalties commensurate with the violations and that escalate for repeat violation. |
| 2.2e. Publicly available listings of violations  The jurisdiction was scored on whether they have a publicly available listing of violations of the alcohol advertising and marketing regulations that includes the name of the advertiser and nature of the violation. | 0.25 | 0.00 = Absence of publicly available listings of violations,  **0.25 = Presence of publicly available listings of violations**. |
| 2.3. Monitoring and public reporting of alcohol industry marketing activities | 1 |  |
| 2.3a. Monitoring of alcohol industry marketing activities    The jurisdiction was scored on whether the authority collects information from the alcohol industry on marketing activities, including expenditures and areas of activity. | 0.5 | **0.00 = No monitoring of alcohol industry marketing activities,**  0.50 = Monitoring the alcohol industry on marketing activities. |
| 2.3b. Public reporting of alcohol industry marketing activities  The jurisdiction was scored on whether, in the interest of transparency, the independent authority makes this information from the alcohol industry on marketing activities public to support evaluation and research. | 0.5 | **0.00 = No public reporting of alcohol industry marketing activities**, 0.50 = Information is publicly available. |
| 3. Impaired Driving Countermeasures | 10 |  |
| 3.1. Impaired driving BAC limits for general population | 4 |  |
| 3.1a. BAC limits for general population (e.g., federal Criminal Code limit at 0.05)  The jurisdiction was scored on whether they had made it a federal criminal offence to drive with a BAC of 0.05% or higher. | 4 | **0.00 = The Criminal Code threshold for driving under the influence is set higher than a BAC of 0.05%,** 4.00 = It is a federal criminal offence to drive with a BAC of 0.05% or higher. |
| 3.2. Impaired driving BAC limits for federally regulated professionals | 1 |  |
| 3.2a. BAC limits for federally regulated professionals  The jurisdiction was scored on whether they had made it a federal criminal offence for federally regulated professionals to operate commercial or passenger vehicles, trains, planes, and boats with a BAC > 0.02%. | 1 | **0.00 = Maximum BAC level for federally regulated commercial drivers is > 0.02% or no applicable Criminal Code provision,** 1.00 = Maximum BAC level for federally regulated commercial drivers is 0.02% or lower. |
| 3.3. Breath testing legislation | 2.5 |  |
| 3.3a. Random breath testing legislation  The jurisdiction was scored on whether they had enacted random breath testing legislation. | 2.5 | 0.00 = No random breath testing legislation, **2.50 = Random breath testing legislation is in place.** |
| 3.4. Evidentiary blood samples | 1.5 |  |
| 3.4a. Evidentiary blood samples  The jurisdiction was scored on whether the police are empowered under the Criminal Code to demand an evidentiary blood sample in any situation in which they are authorized to demand an evidentiary breath sample. | 1.5 | 0.00 = The Criminal Code does not empower police to demand evidentiary blood samples in any situation in which they are authorized to demand an evidentiary breath sample,  **0.50 = The Criminal Code empowers police to demand evidentiary blood samples in some situations in which they are authorized to demand an evidentiary breath sample,** 1.50 = The Criminal Code empowers police to demand evidentiary blood samples in any situation in which they are authorized to demand an evidentiary breath sample. |
| 3.5. Tracking of impaired driving statistics | 1 |  |
| 3.5a. Tracking of impaired driving statistics  The jurisdiction was scored on whether it had a system for the timely, accurate, and comprehensive collection and publishing of alcohol-related transportation deaths and injuries in Canada (including data from all P/Ts). | 1 | 0.00 = No tracking of impaired driving statistics or tracked data are more than four years old, 0.50 = Tracked data do not cover all P/Ts, are not publicly reported or are more than three years old,  **1.00 = Comprehensive data are tracked, publicly reported and are less than three years old.** |
| 4. Health & Safety Messaging | 10 |  |
| 4.1. Status of enhanced alcohol labelling components | 3.50 |  |
| 4.1ai. Legislation for enhanced alcohol labels  The jurisdiction was scored on whether they had legislation in place that would allow for enhanced alcohol labelling components. | 0.30 | **0.00 = No legislation for enhanced alcohol labeling components**, 0.30= Legislation for enhanced alcohol labeling components. |
| 4.1aii. Status of alcohol warning labels  The jurisdiction was scored on whether they had mandatory (i.e., legislated) evidence-based alcohol warning labels, developed independently from the alcohol industry, across a range of topics as a requirement of manufacturer labelling. | 0.95 | +0.20 for warning on alcohol use and cancer risk, +0.15 for warning on alcohol use and health risks, +0.15 for warning on alcohol use by youth and young adults (e.g., MLA laws and health impacts), +0.15 for warning on alcohol use and violence, +0.15 for warning on alcohol use and impaired driving, +0.15 for warning on alcohol use and pregnancy-related risks (e.g., FASD/FAS),  **0.00 = No mandatory evidence-based alcohol warning label on any of the listed topics.** |
| 4.1aiii. Status of standard drink labels  The jurisdiction was scored on whether they had mandatory (i.e., legislated) standard drink information on labels as a requirement of manufacturer labelling. | 0.95 | **0.00 = No standard drink information on alcohol containers**,  0.95 = Legislated standard drink information on alcohol containers. |
| 4.1aiv. Status of national alcohol guidance labels  The jurisdiction was scored on whether they had mandatory (i.e., legislated) national alcohol guidance information on labels as a requirement of manufacturer labelling. | 0.95 | **0.00 = No guidance information on alcohol containers**,  0.95 = Guidance information on alcohol containers. |
| 4.1av. Status of calorie labels  The jurisdiction was scored on whether they had mandatory (i.e., legislated) calorie information on labels as a requirement of manufacturer labelling. | 0.35 | **0.00 = No calorie information on alcohol containers**,  0.35 = Calorie information on alcohol containers. |
| 4.2. Quality of enhanced alcohol labelling components | 3.5 |  |
| 4.2a. Adequacy of label messages  The jurisdiction was scored on whether they had at least one enhanced alcohol labeling component that contained an adequate message that would support consumers in making an informed health decision regarding use of the product. | 1.25 | **0.00 = Label messaging is inadequate in supporting individuals to make an informed choice**, 1.25 = Label messaging is adequate in supporting individuals to make an informed choice. |
| 4.2b. Rotation of warning messages  The jurisdiction was scored on whether their health warning messages on alcohol labels rotated across all alcohol products at least annually in such a way that warning messages could not be selectively applied to certain products. Note: standard drink information, national alcohol guidance information and calorie information should be applied to all beverage products. | 0.75 | **0.00 = Label messaging is not rotating or no enhanced label component,** 0.75 = Label incorporates rotating messaging. |
| 4.2c. Use of pictorials on labels  The jurisdiction was scored on whether they had at least one enhanced alcohol labelling component that was supported by a pictorial such as an image, graphics, icon, etc. | 0.25 | **0.00 = Label does not include graphics or no enhanced label componen**t, 0.25 = Label incorporates graphics. |
| 4.2d. Prominence of labels  The jurisdiction was scored on whether they had at least one enhanced labelling component that was displayed prominently using contrasting colours, occupying at minimum 30% of the display panel and legible under customary conditions of purchase and use (e.g., a minimum of 6-point font). Prominence was specifically defined as front-of-package for alcohol warning messages. | 1.25 | **0.00 = Label messaging is not prominent or no enhanced label component,** 1.25 = Label messaging is prominent. |
| 4.3. Comprehensiveness of health and safety messaging | 3 |  |
| 4.3ai. Public health media campaigns specific to alcohol  The jurisdiction was scored based on whether Health Canada developed/ran or funded on-going annual health and safety media campaigns specific to alcohol, beyond the holiday season which were developed free of industry involvement and contained adequate health messaging (e.g., PSAs disseminated via mainstream media). | 1.4 | **0.00 = No annual media campaigns specific to alcohol or campaign involved industry or contained inadequate messaging,** 1.40 = Annual public health media campaigns specific to alcohol that were developed independently from the alcohol industry and contain adequate health messaging. |
| 4.3aii. Comprehensiveness of the Health Canada health and safety campaign  Jurisdiction was scored on the variation in health and safety topics included in the health and safety campaign. | 0.6 | +0.15 for warning on alcohol use and cancer risk, +0.09 for warning on alcohol use and health risks, +0.09 for warning on alcohol use by youth and young adults (e.g., MLA laws and health impacts), +0.09 for warning on alcohol use and violence, +0.09 for warning on alcohol use and impaired driving, +0.09 for warning on alcohol use and pregnancy-related risks (e.g., FASD/FAS),  **0.00 = No annual health and safety campaign specific to alcohol.** |
| 4.3b. Health Canada alcohol-specific website content  The jurisdiction was scored on whether the primary Health Canada website (e.g., Canada.ca) included a range of evidence-based information related to risks of alcohol use. | 1 | **+ 0.195 for alcohol use and cancer risk, + 0.115 for alcohol use and health risks, + 0.115 for alcohol use among youth and young adults (MLA laws and health impacts), +0.115 for alcohol use and violence, + 0.115 for alcohol use and impaired driving, + 0.115 for alcohol use and pregnancy-related risks (FASD/FAS),**  **+0.115 for treatment resources, +0.115 for national alcohol guidance,**  0.00 = None of the listed topics are included. |
| 5. Physical Availability | 10 |  |
| 5.1. Government controls on commercial alcohol imports | 7 |  |
| 5.1a. Controls on commercial imports  Jurisdiction was scored on whether commercial alcohol products from outside Canada are required by law to be imported by a government authority. | 7 | 0.00 = No legislation requiring all commercially imported alcohol be imported exclusively via government authority,  3.50 = Commercial alcohol must be imported via government authority as inscribed in legislation but with exceptions,  **7.00 = All commercial alcohol must be imported exclusively via government authority as inscribed in legislation.** |
| 5.2 Government controls on personal alcohol imports | 3 |  |
| 5.2a. Controls on personal imports  The jurisdiction was scored on whether they set maximum duty exempt personal alcohol import volumes for absences greater than 48 hours that effectively discourage cross border shopping (e.g., no duty-free alcohol for absence less than 48 hours and a maximum of 1.14L of spirits, 1.5L wine and 8.5L beer for absences exceeding 48 hours). | 3 | 0.00 = No restrictions on duty free personal alcohol import volumes or import volumes set to a level that could encourage cross-border shopping, **3.00 = Duty free personal import volumes are set to effectively discourage cross-border shopping.** |
| 6. Alcohol Control System | 10 |  |
| 6.1. Federal Alcohol Act | 1 |  |
| 6.1a. Intent/mandate of the Federal Alcohol Act  Jurisdiction was scored on whether the intent of Federal Alcohol Act includes explicit mandate/intent related to protection of public health. | 1 | **0.00 = No stated public health mandate in Alcohol Act,**  1.00 = Stated public health mandate in Alcohol Act. |
| 6.2. Comprehensiveness of Federal Alcohol Act | 3.5 |  |
| 6.2a1-a10. Components of Federal Alcohol Act  The jurisdiction was scored on whether they had an Alcohol Act that included evidence-based policy areas: a1. Pricing and taxation a2. Marketing and advertising controls  a3. Impaired driving countermeasures  a4. Health and safety messaging a5. Physical availability a6. Alcohol control system  a7. Minimum legal age a8. National alcohol strategy a9. Screening and treatment interventions  a10. Monitoring and reporting | 3.5 | Federal Alcohol Act that includes the following policy areas:  +0.77 for pricing and taxation, +0.51 for marketing and advertising controls, +0.35 for impaired driving countermeasures, +0.34 for health and safety messaging, +0.32 for physical availability, +0.31 for control system, +0.27 for minimum legal age, +0.23 for national alcohol strategy, +0.22 for screening and treatment interventions, +0.19 for monitoring and reporting, **0.00 = No federal Alcohol Act**  Note: Scores reflect the CAPE domain weights; total points up to a maximum of 3.5 |
| 6.3. Federal government control over alcohol | 3.5 |  |
| 6.3a. Preserving P/T government alcohol monopolies (e.g., financial incentives or tax breaks)  The jurisdiction was scored on whether they provide federal incentives, such as tax breaks or other financial incentives, or other measures for maintaining government control over the retail sale and distribution of alcohol at the provincial/territorial level. | 1.5 | **0.00 = No federal incentives to encourage P/T government control of the distribution and sale of alcohol,** 1.50 = Federal incentives to encourage P/T government control of the distribution and sale of alcohol. |
| 6.3b. Trade law exemptions  The jurisdiction was scored on whether there are trade law exemptions, including those specifically for alcohol, that are permitted in the interests of protecting public health and safety. Note: focused on NAFTA. | 1.5 | 0.00 = No trade law exemptions to protect public health and safety, **0.75 = Trade law exemptions do exist in order to protect public health and safety,**  1.50 = Trade law exemptions, specific to alcohol, exist in order to protect public health and safety. |
| 6.3c. Control of Duty-Free alcohol outlets  The jurisdiction was scored on the proportion of Duty-Free outlets that were government licensed, owned, and run, versus government licensed and privately owned and run. | 0.5 | The jurisdiction was scored on the proportion of Duty-Free outlets that were government licensed, owned, and run, versus government licensed and privately owned and run, up to a maximum of 0.5 points.^[[7]](#footnote-7)^ |
| 6.4. Public health-informed policy decisions | 2 |  |
| 6.4a. Public health input on alcohol policy  The jurisdiction was scored on whether there is legislation requiring public health guidance or input from the ministry of health/public health stakeholders for decision-making and legislative changes around alcohol policies. | 1 | **0.00 = No legislation requiring health/public health involvement,** 1.00 = Legislation requiring health/public health involvement in decision-making and legislative changes around alcohol policies. |
| 6.4b. Targeted public consultation  The jurisdiction was scored on whether there is a formal process for engaging underrepresented groups in public consultation pertaining to alcohol policy changes (e.g., a process for engaging non-industry stakeholder groups including outreach for public input to any priority group beyond industry stakeholders). | 0.6 | **0.00 = No targeted public engagement,** 0.60 = Targeted public engagement for engaging stakeholders beyond industry groups. |
| 6.4c. Transparency of lobbying by alcohol industry  Jurisdiction was scored on whether they had a legislated centralized online public reporting system for industry lobbying activities at the federal level that is geared to the lay public. | 0.4 | 0.00 = No centralized, user-friendly public reporting of industry lobbying, **0.40 = Industry lobbying activities are accessible and transparently reported in centralized online platform.** |
| 7. Minimum Legal Age (MLA) | 10 |  |
| 7.1. Federal legal age for the sale of alcohol | 9 |  |
| 7.1a. Level of legal age for the sale of alcohol  The jurisdiction was scored on the level of the minimum age they set for individuals to whom alcohol can be lawfully sold alcohol under the Criminal Code. | 9 | **0.00 = No minimum age or a minimum age of 17 or younger,**  3.00 = 18 years*, 5.00 = 19 years*, 7.00 = 20 years*,  9.00 = 21 years. *An additional 1.00 point was given if the jurisdiction strengthened minimum age policies by granting graduated access to alcohol by way of stepped restrictions based on strength of alcohol, volume of alcohol or hours of availability. |
| 7.2. Level of legal age for alcohol sales on federally controlled areas | 1 |  |
| 7.2a. Level of legal age for the sale of alcohol on federally controlled areas  The jurisdiction was scored on the level of the minimum age for individuals to whom alcohol can be lawfully sold under the Criminal Code on federally controlled land/waters. | 1 | **0.00= No minimum age or a minimum age of 17 or younger**,  0.25 = 18 years*, 0.50 = 19 years*, 0.75 = 20 years*,  1.00 = 21 years. *An additional 0.125 points were given if the jurisdiction strengthened minimum age policies by granting graduated access to alcohol by way of stepped restrictions based on strength of alcohol, volume of alcohol or hours of availability on federally controlled lands/waters. |
| 8. National Alcohol Strategy | 10 |  |
| 8.1 Status of the national alcohol strategy | 3 |  |
| 8.1a. Status of national alcohol strategy  The jurisdiction was scored on whether they had an alcohol focused public facing strategy or action plan that addresses alcohol as a public health issue. The jurisdiction was scored against an ideal of a standalone national alcohol strategy/action plan that was developed independently from the alcohol industry. | 3 | **0.00 = No national strategy that includes alcohol or the strategy was drafted by/with industry,** 1.50 = A national addictions, mental health, public health, or other strategy that includes alcohol that was not drafted by/with industry,  3.00 = A standalone national alcohol strategy or action plan that was not drafted by/with industry. |
| 8.2. Comprehensiveness of the alcohol strategy | 4 |  |
| 8.2a1-a10. Components of alcohol strategy  The jurisdiction was scored on whether the above-mentioned strategy included a wide range of evidence-based alcohol policy interventions and recommendations (i.e., CAPE P/T policy domains)  a1. Pricing and taxation  a2. Physical availability a3. Alcohol control system a4. Impaired driving countermeasures a5. Marketing and advertising controls a6. Minimum legal age a7. Health and safety messaging a8. Liquor law enforcement a9. Screening and treatment interventions  a10. Monitoring and reporting. | 4 | **+ 0.82 for pricing and taxation recommendations, + 0.71 for physical availability recommendations, + 0.39 for alcohol control system recommendations, + 0.38 impaired driving countermeasures recommendations,**  **+ 0.38 for marketing and advertising controls recommendations, + 0.32 for minimum legal age recommendations, + 0.31 for health and safety messaging recommendations, + 0.28 for liquor law enforcement recommendations, + 0.24 for screening and treatment interventions recommendations, + 0.18 for monitoring and reporting recommendations,**  0.00 = None of the listed recommendations are included.  Note: Scores reflect the CAPE P/T domain weights; total points up to a maximum of 4 |
| 8.3. Implementation of national alcohol strategy | 3 |  |
| 8.3a. Federal strategy funding  The jurisdiction was scored on whether there are dedicated federal funds to develop a fully funded national alcohol strategy. | 0.75 | **0.00 = No national alcohol strategy, funding from industry, or strategy is not funded,** 0.375 = Partial federal funding or funding is part of a larger addictions, mental health, or other strategy funding portfolio (e.g., no project/activity funding),  0.75 = Strategy is fully federally funded. |
| 8.3b. Federal strategy leadership  The jurisdiction was scored on whether the National Alcohol Strategy has an identified leader (individual position or working group) to implement the strategy. Strategy leadership must be free of alcohol industry involvement. | 0.75 | **0.00 = No strategy, no identified strategy leader or strategy leadership includes membership form the alcohol industry**,  0.75 = Has an identified leader to implement the strategy and the leader is independent from the alcohol industry. |
| 8.3ci. Implementation timeline  The jurisdiction was scored on whether they had an implementation timeline for their strategy (e.g., 5-year timeline) that was set independently from the alcohol industry. | 0.375 | **0.00 = No strategy, no implementation timeline or the implementation timeline was set by/with industry,** 0.375 = Jurisdiction has an implementation timeline for their strategy. |
| 8.3cii. Implementation assessment  The jurisdiction was scored on the rigor with which they monitor and publicly report the implementation of the national alcohol strategy. The jurisdiction was scored against an ideal of an on-going implementation assessment that examines implementation through the life of the strategy, beyond one time point in time. Implementation assessments must be free of industry involvement and publicly reported in order to be scored. | 0.375 | **0.00 = No strategy, no public facing assessment(s) or assessment plan, or the assessment plan involves industry**,  0.1875 = There is a one-time public facing implementation assessment or assessment plan for the alcohol strategy that is free of industry involvement,  0.375 = There is regular or reoccurring public facing implementation assessments of the alcohol strategy (e.g., every 3 or 5 years) that are free of industry involvement. |
| 8.3d. Alcohol strategy endorsement  The jurisdiction was scored on whether the alcohol strategy was federally endorsed. | 0.75 | **0.00 = Strategy not endorsed,** 0.75 = Strategy is endorsed by federal government. |
| 8.3e. Penalty – Recency of alcohol strategy  The jurisdiction was scored on how recently the national alcohol strategy had been developed or updated. | -1 | 0.00 points were deducted from the total score for implementation of the strategy if the strategy was created or updated in the past 5 years, 0.50 points were deducted from the total score for implementation of the strategy if the strategy was developed or last updated 6-9 years ago, 1.00 point was deducted from the total score for implementation of the strategy if the strategy was developed or last updated 10 or more years ago.^[[8]](#footnote-8)^ |
| 9. Screening and Treatment Interventions | 10 |  |
| 9.1. National alcohol guidance | 3 |  |
| 9.1a. Development of national alcohol guidance  The jurisdiction was scored on whether there is federal funding to support the development of national guidance on alcohol and health developed independently from alcohol industry interests. | 1.75 | 0.00 = No funding to develop guidance or industry involved in development of guidance, **1.75 = Federal funding available for guidance developed independent of industry.** |
| 9.1b. Endorsement of national alcohol guidance  The jurisdiction was scored on whether national alcohol guidance, developed free from industry, was endorsed at the federal level. | 1.25 | **0.00 = Guidance not endorsed at federal level,**  1.25 = Guidance endorsed at federal level. |
| 9.2. Federal funding for SBIR initiatives at P/T level | 2 |  |
| 9.2a. Federal support for P/T SBIR initiatives  The jurisdiction was scored on whether they provide funding for provincial/territorial level SBIR initiatives either specifically or as part of a comprehensive mental health or problematic substance package (e.g., transfers of health funds). | 2 | 0.00 = No federal funding available, **2.00 = Federal funding available to provinces and/or territories for alcohol SBIR initiatives.** |
| 9.3. Federal SBIR initiatives | 1.4 |  |
| 9.3ai. SBIR initiatives for corrections populations  The jurisdiction was scored on whether they conduct SBIR within populations under federal administration, such as corrections populations. | 0.7 | 0.00 = No tools, 0.35 = General counselling programs only,  **0.70 = Alcohol specific SBIR initiatives**. |
| 9.3aii. SBIR initiatives for military populations  The jurisdiction was scored on whether they conduct SBIR within populations under federal administration, such as military populations. | 0.7 | 0.00 = No tools, 0.35 = General counselling programs only,  **0.70 = Alcohol specific SBIR initiatives.** |
| 9.4. Federal funding for treatment initiatives | 2 |  |
| 9.4a. Federal support for P/T treatment initiatives  The jurisdiction was scored on whether they have earmarked funds for supporting alcohol-specific treatment at the provincial and territorial level (e.g., health transfer funds). | 2 | **0.00 = No earmarked funds,** 2.00 = Earmarked funds for supporting alcohol treatment at P/T level. |
| 9.5. Federal treatment initiatives | 1.4 |  |
| 9.5ai. Treatment services for corrections populations  The jurisdiction was scored on whether they provide treatment services within populations under federal administration, such as corrections populations (beyond 12-step peer-to-peer model). | 0.7 | 0.00 = No treatment services, **0.70 = Alcohol-specific treatment services beyond 12-step peer-to-peer model.** |
| 9.5aii. Treatment services for military populations  The jurisdiction was scored on whether they provide treatment services within populations under federal administration, such as military populations (beyond 12-step peer-to-peer model). | 0.7 | 0.00 = No treatment services, **0.70 = Alcohol-specific treatment services beyond 12-step peer-to-peer model.** |
| 9.6. Federal tracking and reporting of SBIR and treatment initiatives | 0.2 |  |
| 9.6a. Tracking and reporting of SBIR and treatment for federal populations  The jurisdiction was scored on whether they conduct tracking and reporting of SBIR and treatment for populations under federal administration such as corrections and military. | 0.2 | 0.00 = No tracking or reporting of SBIR and treatment,  **0.10 = Tracking and reporting of one population,** 0.20 = Tracking and reporting of both populations. |
| 10. Monitoring and Reporting | 10 |  |
| 10.1 Comprehensiveness of national alcohol monitoring program | 4 |  |
| 10.1ai. Alcohol consumption indicators  The jurisdiction was scored on whether they provide federal funding for a national alcohol monitoring program that tracks alcohol consumption by sales and survey data. | 0.5 | 0.00 = No funding for reporting activities,  0.25 = Partially funded, **0.50 = Comprehensively funded.** |
| 10.1aii. Morbidity indicators  The jurisdiction was scored on whether they provide federal funding for a national alcohol monitoring program that tracks alcohol-related morbidity indicators. | 0.5 | 0.00 = No funding for reporting activities,  0.25 = Partially funded, **0.50 = Comprehensively funded.** |
| 10.1aiii. Mortality indicators  The jurisdiction was scored on whether they provide federal funding for a national alcohol monitoring program that tracks alcohol-related mortality indicators. | 0.5 | 0.00 = No funding for reporting activities,  0.25 = Partially funded, **0.50 = Comprehensively funded.** |
| 10.1aiv. Crime indicators  The jurisdiction was scored on whether they provide federal funding for a national alcohol monitoring program that tracks alcohol-related crime indicators. | 0.5 | 0.00 = No funding for reporting activities,  0.25 = Partially funded, **0.50 = Comprehensively funded.** |
| 10.1av. Cost indicators  The jurisdiction was scored on whether they provide federal funding for a national alcohol monitoring program that tracks alcohol-related cost indicators. | 0.5 | 0.00 = No funding for reporting activities,  0.25 = Partially funded, **0.50 = Comprehensively funded.** |
| 10.1avi. Policy change indicators  The jurisdiction was scored on whether they provide federal funding for a national alcohol monitoring program that tracks policy change indicators and the heath impacts of these changes. | 0.5 | 0.00 = No funding for reporting activities,  0.25 = Partially funded, **0.50 = Comprehensively funded.** |
| Bonus: comprehensiveness synergy | 1 | Synergy points: An additional 1.0 points for a comprehensive monitoring program that captures all 6 alcohol indicators. The synergy score was in direct proportion to the number of indicators that they fund.^[[9]](#footnote-9)^ |
| 10.2. Accessibility of reporting | 3 |  |
| 10.2ai. Transparency of reporting - alcohol consumption indicators  The jurisdiction was scored based on the degree to which the results were made public. | 0.25 | 0.00 = No reporting,  **0.25 = Public reporting.** |
| 10.2aii. Transparency of reporting - morbidity indicators    The jurisdiction was scored based on the degree to which the results were made public. | 0.25 | 0.00 = No reporting,  **0.25 = Public reporting.** |
| 10.2aiii.Transparency of reporting - mortality indicators  The jurisdiction was scored based on the degree to which the results were made public. | 0.25 | 0.00 = No reporting,  **0.25 = Public reporting.** |
| 10.2aiv.Transparency of reporting - crime indicators  The jurisdiction was scored based on the degree to which the results were made public. | 0.25 | 0.00 = No reporting,  **0.25 = Public reporting.** |
| 10.2av.Transparency of reporting - cost indicators  The jurisdiction was scored based on the degree to which the results were made public. | 0.25 | 0.00 = No reporting,  **0.25 = Public reporting.** |
| 10.2avi.Transparency of reporting - policy change  The jurisdiction was scored based on the degree to which the results were made public. | 0.25 | 0.00 = No reporting,  **0.25 = Public reporting.** |
| Bonus: comprehensiveness synergy | 0.5 | An additional 0.50 points awarded for a comprehensive monitoring program that captures all 6 alcohol indicators. The synergy score was in direct proportion to the number of indicators that they publicly report.^[[10]](#footnote-10)^ |
| 10.2bi. Frequency of reporting - alcohol consumption indicators  The jurisdiction was scored on whether they provide reporting at regular intervals on alcohol consumption by sales and survey data. | 0.125 | 0.00 = No reporting or reporting every 5 years+,  0.10 = Reporting every 2-4 years, **0.125 = Reported annually.** |
| 10.2bii. Frequency of reporting - morbidity indicators    The jurisdiction was scored on whether they provide reporting at regular intervals on alcohol-related morbidity indicators. | 0.125 | 0.00 = No reporting or reporting every 5 years+,  **0.10 = Reporting every 2-4 years,** 0.125 = Reported annually. |
| 10.2biii. Frequency of reporting - mortality indicators  The jurisdiction was scored on whether they provide reporting at regular intervals on alcohol-related mortality indicators. | 0.125 | 0.00 = No reporting or reporting every 5 years+,  0.10 = Reporting every 2-4 years, **0.125 = Reported annually.** |
| 10.2biv. Frequency of reporting - crime indicators  The jurisdiction was scored on whether they provide reporting at regular intervals on alcohol-related crime indicators. | 0.125 | 0.00 = No reporting or reporting every 5 years+,  0.10 = Reporting every 2-4 years, **0.125 = Reported annually.** |
| 10.2bv. Frequency of reporting - cost indicators  The jurisdiction was scored on whether they provide reporting at regular intervals on alcohol-related cost indicators. | 0.125 | 0.00 = No reporting or reporting every 5 years+,  **0.10 = Reporting every 2-4 years,** 0.125 = Reported annually. |
| 10.2bvi. Frequency of reporting - policy change  The jurisdiction was scored on whether they provide reporting at regular intervals on policy change indicators. | 0.125 | 0.00 = No reporting or reporting every 5 years+,  **0.10 = Reporting every 2-4 years,** 0.125 = Reported annually. |
| Bonus: comprehensiveness synergy | 0.25 | An additional 0.25 points awarded for a comprehensive monitoring program that captures all 6 alcohol indicators. The synergy score was in direct proportion to the number of indicators that they report on annually.^[[11]](#footnote-11)^ |
| 10.3. Leadership for alcohol monitoring and reporting | 3 |  |
| 10.3ai. Central reporting  The jurisdiction was scored on whether they implemented public centralized reporting for alcohol-related indicators (e.g., alcohol consumption, harms, costs, and policies). | 1 | **0.00 = No centralized system,** 1.00 = Central public database or reporting system. |
| 10.3aii. Leadership for alcohol monitoring and reporting    The jurisdiction was scored on whether there was an identifiable leader responsible for monitoring and reporting alcohol-related harm and consumption. | 1 | 0.00 = No organization, committee or individual leader identified, **1.00 = Readily identifiable leadership.** |
| 10.3aiii. Knowledge translation in past two years  The jurisdiction was scored on whether there have been any active KT activities on alcohol indicators. | 1 | 0.00 = No knowledge products or activities in the past two years, **1.00 = Active knowledge translation in the past two years.** |

1. Numbers may not sum correctly due to rounding to two decimal places. [↑](#footnote-ref-1)
2. Scores within each indicator were summed and divided by the maximum possible score to determine the final domain percentage score. Scores were also converted to letter grades according to the following criteria: A+ = 90-100%; A = 85-89%; A- = 80-84%; B+ = 77-79%; B = 73-76%; B- = 70-72%; C+ = 67-69%; C = 63-66%; C- = 60-62%; D+ = 57-59%; D = 53-56%; D- = 50-52%; F = 0-49% [↑](#footnote-ref-2)
3. **1.153575 points** were awarded for this indicator. Excise tax rates for high strength spirits meet recommendations, and high strength spirits represent 46.143% of the ethanol market share (46.143% of 2.5 points is 1.153575). [↑](#footnote-ref-3)
4. **1.153575 points** were awarded for this indicator. There are volumetric excise taxes for high-strength spirits. High strength spirits represent 46.143% of the ethanol market share (46.143% of 2.5 points is 1.153575). [↑](#footnote-ref-4)
5. **0.08188867 points** were awarded for this indicator. 32.7554681% of federal alcohol taxes are collected by volumetric excise taxation (32.7554681% of 0.25 is 0.08188867). [↑](#footnote-ref-5)
6. While indicators 2.1a-d each received 0.00 points, the CRTC code qualifies as a marketing restriction. [↑](#footnote-ref-6)
7. **0.00 points** were awarded for this indicator. All Duty-Free outlets are government licensed, but privately owned and run. [↑](#footnote-ref-7)
8. **0.00 points** were awarded for this indicator. While the criteria for a 1.00-point reduction were met, overall indicator scores cannot be negative, so as to not carry over to other indicators. [↑](#footnote-ref-8)
9. **1.00 point** was awarded for this indicator. The federal government provides comprehensive funding for all six indicators. [↑](#footnote-ref-9)
10. **0.50 points** were awarded for this indicator. All six indicators are publicly reported. [↑](#footnote-ref-10)
11. **0.125 points** were awarded for this indicator. Three of six indicators are reported on annually. [↑](#footnote-ref-11)
